# Grundlagen der Mikrobiomforschung

**DOI:** 10.1007/s00106-025-01656-7

**Published:** 2025-08-13

**Authors:** Julia Eckl-Dorna, Petra Pjevac

**Affiliations:** 1 Universitätsklinik für Hals‑, Nasen- und Ohrenkrankheiten, Medizinische Universität Wien, Währinger Gürtel 18–20, 1090 Wien, Österreich; 2 Department für Mikrobiologie und Ökosystemforschung, Zentrum für Mikrobiologie und Umweltsystemwissenschaft, Universität Wien, Djerassiplatz 1, 1030 Wien, Österreich; 3 Joint Microbiome Facility der Medizinischen Universität Wien und der Universität Wien, Djerassiplatz 1, 1030 Wien, Österreich

**Keywords:** Mikroorganismen, 16S-rRNA-Gen, Sequenzierung, Geringe Biomasse, Metagenom, Microorganisms, 16S rRNA gene, Sequencing, Low biomass, Metagenome

## Abstract

Die Mikrobiomforschung im HNO(Hals-, Nasen- und Ohren)-Bereich hat in den letzten Jahrzehnten erheblich an Bedeutung gewonnen. Dabei haben moderne Sequenzierungsmethoden die traditionelle Kultivierung weitgehend ersetzt. Um zuverlässige und vergleichbare Daten zu erhalten, sind standardisierte Vorgehensweisen essenziell. Der Artikel erläutert grundlegende Begriffe wie Mikrobiom (Gesamtheit der Mikroorganismen und ihrer Umgebung) und Mikrobiota (nur lebende Mikroorganismen) sowie die mikrobielle Taxonomie. Wichtige Maße zur Bewertung des Mikrobioms sind die α‑Diversität (Artenreichtum und -verteilung innerhalb einer Probe) und die β‑Diversität (Unterschiede zwischen Proben). Zwei Hauptmethoden zur Mikrobiomsequenzierung werden unterschieden: 1. 16S-rRNA-Gen-Amplikon-Sequenzierung (bestimmt die Zusammensetzung von mikrobiellen Gemeinschaften durch Sequenzierung des PCR(„polymerase chain reaction“)-Produkts eines bestimmten Gens); 2. metagenomische Sequenzierung (sequenziert das gesamte genetische Material einer Probe und ermöglicht tiefergehende Analysen). Da die mikrobielle Biomasse in der Nase gering ist, sind sorgfältige Studiendesigns und Kontrollen unerlässlich. Die Mikrobiomforschung ist ein wachsendes Feld mit großem Potenzial, erfordert jedoch präzise Planung und bioinformatische Expertise für aussagekräftige Ergebnisse.

## Abstract (en)

Microbiome research in the ear nose and throat (ENT) field has substantially gained in importance in recent decades. Modern sequencing methods have largely replaced traditional cultivation. Standardized protocols are essential to ensure reliable and comparable data. The article explains key terms, such as microbiome (the entire microbial community and its environment) and microbiota (only living microorganisms), along with the microbial taxonomy. Important measures for microbiome assessment include the alpha-diversity (species richness and distribution within a sample) and beta-diversity (differences between samples). A differentiation is made between two main approaches to microbiome sequencing: 1) 16S rRNA gene amplicon sequencing (identifies microbial community composition by sequencing the PCR product of a specific gene), 2) metagenomic sequencing (sequences the entire genomic material of a sample, allowing deeper insights). As nasal microbiome biomass is low, careful study design and controls are crucial. Microbiome research is a rapidly growing field with great potential but it requires meticulous planning and bioinformatics expertise for meaningful results.

Die Erforschung des humanen Mikrobioms, nicht nur des Darms, sondern auch anderer Habitate wie des HNO(Hals-Nasen-Ohren)-Bereichs hat in den letzten Jahrzehnten enorm an Bedeutung gewonnen, wobei in der Forschung der klassische „Kultivierungsansatz“ fast gänzlich den modernen Sequenzierungsmethoden gewichen ist. Doch gerade bei Letzteren gilt es, wichtige Grundsätze zu beachten, um qualitätsvolle und international vergleichbare Daten zu erzielen. Dieser Artikel soll einen Überblick über die derzeit gebräuchlichen Konzepte und Technologien sowie deren praktische Umsetzung in der HNO-Forschung geben.

## Grundlegende Terminologie

**Abb. 1:**
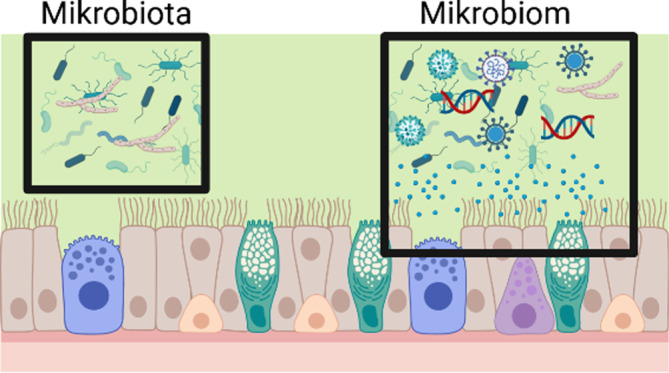
Unterschied zwischen Mikrobiom und Mikrobiota: Als Mikrobiota werden lebende Mikroorganismen bezeichnet, die eine Region bewohnen. Das Mikrobiom umfasst die Mikobiota und deren „Aktionsraum Aktivität“. (Erstellt mit BioRender.com)

- In der medizinischen Forschung werden die Begriffe Mikrobiom und Mikrobiota häufig synonym verwendet, es handelt sich jedoch um unterschiedliche Bezeichnungen. Nichtsdestotrotz ist die Begriffsdefinition in der Mikrobiomforschung keine einfache und häufig kontroversiell diskutiert [1]. Folgende Definition ist derzeit eine häufig verwendete (Abb. 1): Als Mikrobiota werden jene lebenden Mikroorganismen bezeichnet, die eine Region, z. B. des Körpers, bewohnen. Hierzu zählen z. B. Bakterien, Archaeen, Pilze und Einzeller. Der Begriff Mikrobiom hingegen umfasst grundsätzlich das gesamte Habitat, also nicht nur die Gemeinschaft der Mikroorganismen, sondern auch deren „Aktionsraum Aktivität“ („theatre of activity“). Dieser beinhaltet das gesamte Spektrum an Molekülen, die von Mikroorganismen produziert werden, also auch ihre Strukturelemente wie Nukleinsäuren oder Polysaccharide, Metabolite sowie Moleküle, die von koexistierenden Wirten produziert oder durch die Umweltbedingungen gegeben sind. Daher sind alle mobilen genetischen Elemente wie Phagen oder Viren (auch häufig als Virom bezeichnet) vom Begriff Mikrobiom umfasst, zählen aber nicht zu den Mikrobiota.
- Unter Metagenom versteht man die Sammlung aller genomischen Informationen, die in einer Probe enthalten sind. Seine Gesamtheit kann in einer Probe durch die unten beschriebene metagenomische Sequenzierung analysiert werden kann

## Mikrobielle Taxonomie

Als Taxon wird eine Gruppe von Lebewesen bezeichnet, die sich anhand gemeinsamer Merkmale von anderen Organismen unterscheiden. Üblicherweise werden Bakterien und Archaeen in folgendem hierarchischen System klassifiziert: Zuoberst steht das Phylum, es folgen Klasse, Ordnung, Familie, Genus und zuletzt Spezies. So wird *Staphylococcus aureus*, ein im Respirationstrakt häufig vorkommendes pathogenes Bakterium, als Beispiel in Tab. 1 in die Bakterienklassifikation eingeordnet:

**Tab. 1:** Taxonomische Klassifizierung von *Staphylococcus aureus*

Rang oder Level	Taxonomischer Name
Phylum/Stamm	Bacillota
Klasse	*Bacilli*
Ordnung	*Bacillales*
Familie	*Staphylococcaceae*
Genus/Gattung	*Staphylococcus*
Spezies/Art	*Staphylococcus aureus*

## Maßeinheiten zur Beurteilung des Mikrobioms

**Abb. 2:**
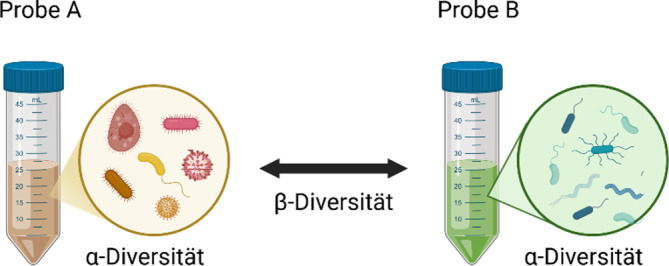
α- und β‑Diversität: α‑Diversität ist ein Maß für Artenreichtum und -gleichheit innerhalb einer Probe, während die β‑Diversität die Variabilität zwischen den unterschiedlichen Proben misst. (Erstellt mit BioRender.com)

Häufig werden in wissenschaftlichen Publikationen die Unterschiede im Mikrobiom mittels α‑ und β‑Diversität angegeben [2]. Grob gesagt, misst die α‑Diversität die Anzahl und die relative Häufigkeit verschiedener Arten, die in einem Organismus oder innerhalb einer Probe nachgewiesen werden können (sog. Punktdiversität), und sie wird häufig mit dem Chao-I-, dem Shannon-Wiener- oder dem Simpson-Index angegeben. Diese 3 unterschiedlichen Indizes berücksichtigen in unterschiedlichem Maße die „richness“ (Artenreichtum innerhalb einer Probe) und die „evenness“ (Artengleichverteilung innerhalb einer Probe) der verschiedenen Spezies innerhalb eines Samples. Die β‑Diversität hingegen misst die Variabilität zwischen den einzelnen Proben oder Individuen im gleichen Lebensraum und stellt somit ein Maß für die Unterschiede in der Artenvielfalt dar (Abb. 2). Sie wird meist verwendet, um die Differenz in der Mikrobiomzusammensetzung zwischen 2 Gruppen darzustellen und wird mithilfe der Aitchison-, der Bray-Curtis- oder der UniFrac-Distanz dargestellt.

## Meilensteine in der Mikrobiomforschung

Bereits im Jahr 1676 hat Antony van Leeuwenhoek mittels seines selbstgebauten Mikroskops die ersten Bakterien in einem Mundabstrich beobachtet [3]. Fasziniert von ihrer Beweglichkeit in Flüssigkeiten nannte er diese Organismen „kleine Tiere“ oder „Animalcules“. Sogar ihre Länge konnte er mit 3 µm genau bestimmen, und er glaubte, dass einige von ihnen ohne Luft leben könnten. Er war auch der Erste, der *Giardia* in seiner eigenen Stuhlprobe beschrieb. Es dauerte weitere 2 Jahrhunderte, bis Louis Pasteur 1860 als Erster ein Medium beschrieb, das Wachstum von Bakterien begünstigte, und somit die Grundlage für die Erforschung dieser schaffte. Es folgten die Entwicklung von Agar und sterilen Containern sowie die Charakterisierung der Bakterien anhand des Sauerstoffbedarfs in aerobe und anaerobe Bakterien [4]. Ein weiterer Meilenstein war die Entwicklung der Gram-Färbung durch Hans Christian Gram im Jahr 1884, die die Einteilung der Bakterien aufgrund ihrer Zellwandbeschaffenheit in grampositiv und gramnegativ erschuf. Dies ist aufgrund des unterschiedlichen Gehalts des Bausteins Peptidoglykan in der Bakterienzellwand möglich, der den Farbstoff Gentianaviolett bindet. Bei grampositiven Bakterien enthält die Zellwand eine dicke Schicht an Peptidoglykanen und färbt sich somit dunkelviolett. Aufgrund der geringen Menge an Peptidoglykanen bei gramnegativen Bakterien färben sich diese rötlich.

Bakterienkulturen auf Agarplatten oder in sterilen Behältern waren für viele Jahrzehnte die Methode der Wahl für die Charakterisierung der Bakterien und die Voraussetzung für die Entdeckung von Antibiotika. Limitiert wurde diese Methode nicht nur durch die oft langwierige Prozedur, sondern auch durch die Tatsache, dass viele Mikrobiota, darunter der Großteil der anaeroben Bakterien, nicht kultiviert werden können. 16S-ribosomale Ribonukleinsäure(16S-rRNA)-Gen-Amplikon-basierte und Metagenomsequenzierungen (ganzes Genom) haben das Feld der Mikrobiomforschung ab den 1990er-Jahren revolutioniert. Hierzu trug auch die Einführung der High-throughput-Methoden NGS („next generation sequencing“) ab den 2010er-Jahren maßgeblich bei. Die moderne Mikrobiomforschung ist relativ kostengünstig und ermöglicht nicht nur Einblicke in Komposition und Diversität des Mikrobioms, sondern auch funktionelle Analysen wie z. B. die Bestimmung der Koevolution des Mikrobioms mit seinem Wirt. So gewinnt das Mikrobiom als „verborgenes Organ“, dessen Komposition und Interaktion mit dem Immun‑, Endokrin- und Nervensystem für die Physiologie des Körpers eine wichtige Rolle spielt, zunehmende Anerkennung [5].

## Methoden zur Mikrobiomsequenzierung

**Abb. 3:**
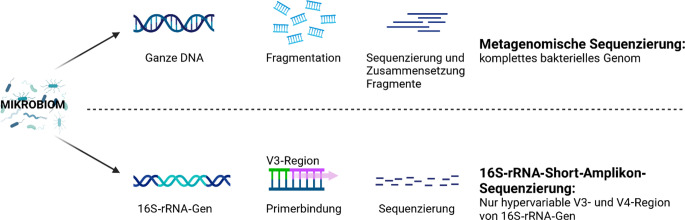
Methoden der Mikrobiomsequenzierung: Bei der metagenomischen Sequenzierung wird das gesamte genomische Material einer Probe sequenziert, während bei der *16S-rRNA*(16S-ribosomale Ribonukleinsäure)-Gen-Amplikon-Sequenzierung mithilfe spezifischer Primer die Zusammensetzung der mikrobiellen Gemeinschaft auf Basis des 16S-rRNA-Gens bestimmt wird. (Erstellt mit BioRender.com)

Grob unterscheidet man 2 Hauptmethoden zur Mikrobiomsequenzierung, nämlich die gezielte Amplikonsequenzierung, wo ein bestimmtes Gen mit Primern amplifiziert wird, und die Metagenomsequenzierung, bei der die gesamte vorhandenen genetische Information analysiert wird (Abb. 3).

### Amplikonsequenzierung

Die Methode der Amplikonsequenzierung beruht auf der Bindung von universellen Primern (kurze einzelsträngige DNA-Abschnitte [18–25 Basen lang], die als Startpunkt für die DNA-Polymerase bei der PCR [„polymerase chain reaction“] dienen) an hoch konservierte Regionen im Genom von ausgewählten Mitgliedern des Mikrobioms und auf der Sequenzierung der daraus resultierenden PCR-Produkte. Universelle Primer sind für Archaeen und Pilze beschrieben worden, am häufigsten wird jedoch das bakterielle 16S-rRNA-Gen für die Sequenzierung von Bakterien herangezogen. Bereits in den 1960er-Jahren begann Carl Woese, das 16S-rRNA-Gen, das für eine Komponente der 30S-Untereinheit des Ribosoms in Prokaryoten kodiert und somit in allen Bakterien und Archaeen vorhanden ist, zu analysieren [6]. Er entdeckte, dass dieses Gen, das aus etwa 1500 Basenpaaren besteht, sowohl hoch konservierte als auch variable Regionen besitzt und sich daher gut für die kulturunabhängige Bestimmung der Phylogenie eignet. Die Primer werden so designt, dass sie an eine konservierte Region im 16S-rRNA-Gen binden. Die darauffolgende variable Region enthält artenspezifische Sequenzunterschiede, die dabei helfen, die Bakterien und Archaeen voneinander zu unterscheiden. Besonders häufig werden die Regionen V3–V4 oder V4 zur Analyse genutzt, da sie eine gute Balance zwischen Variabilität und Sequenzlänge bieten.

Die derzeit am häufigsten verwendete Technik ist die 16S-rRNA-Gen-Amplikon-Short-read-Sequenzierung mithilfe von NGS, da sie rasch einen guten Überblick über die Zusammensetzung des Mikrobioms liefert [7]. Diese High-throughput Methode erlaubt die gleichzeitige Sequenzierung einer großen Anzahl kurzer mikrobieller DNA-Fragmente (150–400 Basenpaare). So lassen sich die Bakterien und Archaeen meist bis zum Genuslevel charakterisieren. Die Sequenzen werden, basierend auf ihrer Ähnlichkeit, meist mithilfe von 16S-rRNA-Gen-Referenzdatenbanken abgeglichen. Hierfür werden sie zuvor – je nach Analysepipeline – in OTU („operational taxonomic units“) oder ASV („amplicon sequence variants“) eingeteilt. Bei den OTU werden alle Sequenzen, die über 97 % Ähnlichkeit aufweisen, in einer OTU zusammengefasst. Diese Form der Einteilung hat jedoch den Nachteil, dass eine Sequenz zu mehr als nur 1 anderen Sequenz mehr als 97 % Ähnlichkeit haben kann und daher verschiedene Analysen nicht miteinander vergleichbar sind, da dieselbe Sequenz fälschlicherweise unterschiedlichen „units“ zugeteilt wird. Dies führte zur Entwicklung der Einteilung in ASV. Hier wird mithilfe statistisch berechneter Fehlerprofile versucht, zwischen biologischer und technisch bedingter Sequenzvariation zu unterscheiden, um Sequenzen genauer zuordnen zu können. Somit haben ASV eine höhere Auflösung sowie eine bessere Sensitivität und Spezifität im Vergleich zu OTU. Wichtig bleibt zu betonen, dass ASV/OTU nicht einer Spezies gleichzusetzen sind – eine bestimmte ASV kann mehrere Spezies beinhalten, und eine Spezies kann durch mehrere ASV vertreten sein, wenn es Sequenzunterschiede zwischen multiplen 16S-rRNA-Gen-Kopien in dieser Spezies gibt.

Nachteile der 16S-rRNA-Gen-Amplikon-Methode sind Ungenauigkeiten in der taxonomischen Zuordnung auf Spezieslevel aufgrund der kurzen sequenzierten Fragmente sowie die große Anzahl an unterschiedlichen Sequenzierung- und Analyseplattformen, die die Vergleichbarkeit der Daten erschwert. Nichtsdestotrotz ist diese Methode auch in Studien im HNO-Bereich weit verbreitet.

Es gibt daher auch die Möglichkeit, 16S-rRNA-Gene in ihrer vollen Länge zu sequenzieren, wobei mittels „shotgun sequencing“ alle DNA-Fragmente einer mikrobiellen Gemeinschaft sequenziert und danach zusammengesetzt werden. Hier kann in die Tiefe, bis zum Spezies- oder sogar Strain-Level, sequenziert werden. So konnte mittels Sequenzierung der Full-length-16S-rRNA bei einigen Kindern mit durch *Mycoplasma pneumoniae* hervorgerufener Lungenentzündung ein starkes Vorhandensein von Koinfektionen mit anderen pathogenen Bakterien wie *Staphylococcus epidermidis, Streptococcus pneumoniae* oder *Pseudomonas aeruginosa* nachgewiesen werden [8].

### Metagenomsequenzierung

Bei der aufwendigeren und kostenintensiveren Methode der metagenomen Sequenzierung werden die DNA-Sequenzen in der Probe fragmentiert, möglichst in ihrer Gesamtheit sequenziert und danach zusammengesetzt. So wird ein genaues Abbild des gesamten genetischen Materials anstatt der Fragmente des 16S-rRNA-Gens erreicht, und somit gelingt es auch, z. B. bakterielle Virulenzfaktoren oder Gene, die mit Antibiotikaresistenzen assoziiert sind, zu identifizieren. Mit dieser Methode wurde im renommierten Journal *Science* im Jahr 1995 erstmals das komplette Genom von *Haemophilus influenzae* und *Mycoplasma genitalium* publiziert [9, 10]. Allerdings ist zu bedenken, dass hier auch Phagen‑, Virus‑, Archaea- oder Pilzgenome sowie Informationen zu anderen Eukaryoten und auch teilweise nukleare und mitochondriale DNA des Wirts mitabgebildet werden. Daher ist eine viel tiefere Sequenzierung nötig, um Informationen zu den relativ seltenen Prokaryoten und ihren vergleichbar kleinen Genomen zu erhalten. Dies verursacht einerseits deutlich höhere Kosten, andererseits auch eine sehr hohe Datenmenge, die gewissenhaft analysiert werden muss. Dies ist mit traditionellen bioinformatischen Analysen und Algorithmen oft schwer zu bewältigen. Daher müssen hier häufig neue Tools unter Zuhilfenahme von Machine Learning entwickelt werden. Zu beachten ist, dass auch mit den fortschrittlichsten bioinformatischen Methoden die Rekonstruktion ganzer mikrobieller Genome aus respiratorischer Schleimhaut aufgrund der niedrigen Biomasse sehr schwer ist.

## Wichtige Überlegungen für die klinische Mikrobiomforschung

Die Biomasse, also die gesamte organische Masse von Lebewesen, ist in der Nase sehr gering, und daher sind die gründliche Planung und Anwendung der nötigen Kontrollen essenziell [11]. Bei der Planung ist auch die Fallzahlplanung mithilfe eines erfahrenen Biostatistikers zu berücksichtigen. Nach erfolgreicher Bewilligung der Studie durch die Ethik ist es von Vorteil, die Studie möglichst von einem kleinen Studienteam durchführen zu lassen, damit die Probenentnahme immer in der gleichen Weise erfolgt. Die Proben sollten auf Eis gelagert und möglichst rasch bei −80 °C tiefgefroren werden. Zur Aufarbeitung und Analyse der Proben empfiehlt sich die Zusammenarbeit mit einem erfahrenen Team aus Mikrobiologen und Bioinformatikern. Aufgrund der hohen Sensitivität der Methode müssen sog. unspezifische Hintergrundsignale abgezogen werden, um ein eindeutiges Patientensignal zu erhalten. Folgende Kontrollen sind z. B. unerlässlich:

1. Sampling Blank Control: Hier können während des Probenentnahmeverfahrens eingebrachte DNA-Kontaminationen, wie etwa durch Wattestäbchen, Konservierungsmittel oder Raumluft, erkannt werden (z. B. Probestäbchen 20 s lang im Raum, wo die Probe entnommen wird, durch die Luft schwenken).
2. DNA Extraction Blank Control: Diese Kontrolle schafft ein Bild der während des Extraktionsprozesses aus Kits oder Reagenzien entstandenen Kontaminationen.
3. No-Template Amplification Control: Dies ist wichtig, um die DNA-Kontamination während der Bibliotheksvorbereitung und der Sequenzierung zu erkennen.

Die RIDE („report methodology/include controls in sequencing/determine the level of contamination to measure the limit of detection/explore the impacts of contamination in downstream analysis“)-Guidelines geben einen ersten guten Überblick zur Problematik der Verarbeitung von Proben mit geringer Biomasse [12]. Auch die Analyse erfordert Erfahrung und sehr gute bioinformatische Kenntnis. Sollte eine Probe – nach Abzug des Hintergrundsignals und der sog. „decontamination“ – weniger als 500–1000 Reads haben, kann diese als statistisch nicht aussagekräftig gewertet werden.

Zusammengefasst ist die sequenzierungsgestützte Mikrobiomforschung ein spannendes und wachsendes Feld, wobei viel Sorgfalt auf Studiendesign und -analyse gelegt werden sollte.

## Fazit für die Praxis

- Die Mikrobiomforschung hat in den letzten Jahrzehnten im HNO-Bereich enorm an Bedeutung gewonnen.
- Die gezielte 16S-rRNA(16S-ribosomale RNA)-Gen-Amplikon-Sequenzierung bietet einen guten Überblick über das vorhandene Mikrobiom.
- Metagenome Sequenzierung erlaubt einen tiefen Einblick auch in funktionelle Charakteristika des Mikrobioms, ist allerdings kostenintensiver und komplexer in der Analyse.
- Bei Durchführung von Studien zum Mikrobiom empfehlen sich
  a. Studium von Leitlinien (z. B. RIDE [„report methodology/include controls in sequencing/determine the level of contamination to measure the limit of detection/explore the impacts of contamination in downstream analysis“])
  b. Zusammenarbeit mit Spezialisten für Mikrobiom inkl. Bioinformatiker
  c. gründliche Fallzahlplanung
  d. geeignete zahlreiche Kontrollen (Luft und Reagenzien)
  e. Ausschluss von Proben, die zu wenig Reads haben bzw. ein zu starkes Hintergrundsignal aufweisen
